# Occupation Times on the Legs of a Diffusion Spider

**DOI:** 10.3390/e27020179

**Published:** 2025-02-08

**Authors:** Paavo Salminen, David Stenlund

**Affiliations:** 1 Faculty of Science and Engineering, Åbo Akademi University, 20500 Åbo, Finland; 2 Department of Mathematics, University of British Columbia, Vancouver, BC V6T 1Z2, Canada; stenlund@math.ubc.ca

**Keywords:** diffusions on graphs, Walsh’s Brownian motion, Green’s function, resolvent, Kac’s moment formula, additive functional, moment generating function, 60J60, 60J55, 60J65, 05A10

## Abstract

We study the joint moments of occupation times on the legs of a diffusion spider. Specifically, we give a recursive formula for the Laplace transform of the joint moments, which extends earlier results for a one-dimensional diffusion. For a Bessel spider, of which the Brownian spider is a special case, our approach yields an explicit formula for the joint moments of the occupation times.

## 1. Introduction

When trying to understand and quantify the behavior of a stochastic process, we are often faced with analyzing various functionals of the process. Such functionals include first passage times to subsets of the state space, maximum (minimum) value up to random/fixed times, and occupation times in subsets. The first question is, of course, whether it is possible to find the distribution of the functional. Unfortunately, this is often not possible, or the expression is too complicated to have any practical value. In some cases, the Laplace transform of the distribution is more tractable for further studies than the distribution itself. In addition, the moments of the distribution often determine the distribution uniquely via a series expansion. Hence, being able to calculate the moments is a good contribution in many respects. In this paper, we study the moments of occupation time functionals for a family of stochastic processes that we call diffusion spiders. We proceed now to explain intuitively what lies behind this notion, to give references to earlier works, and to indicate some applications.

The process known as Walsh Brownian motion was introduced by J.B. Walsh in 1978 as an extension of the skew Brownian motion. The Walsh Brownian motion lives in $R^{2}$, best expressed using polar coordinates. When away from the origin, the angular coordinate stays constant (so the process moves along a line), while the radial distance follows a positive excursion from 0 of a standard Brownian motion. Intuitively and roughly speaking, every time the process reaches the origin, a new angle is randomly selected according to some distribution on [0,2π). This process was brilliantly described by Walsh in the following way [1]:

It is a diffusion which, when away from the origin, is a Brownian motion along a ray, but which has what might be called a roundhouse singularity at the origin: when the process enters it, it, like Stephen Leacock’s hero, immediately rides off in all directions at once.

The construction of the Walsh Brownian motion was described in more detail by Barlow, Pitman, and Yor [2]. We also refer to Salisbury [3] and Yano [4].

**Figure 1:**
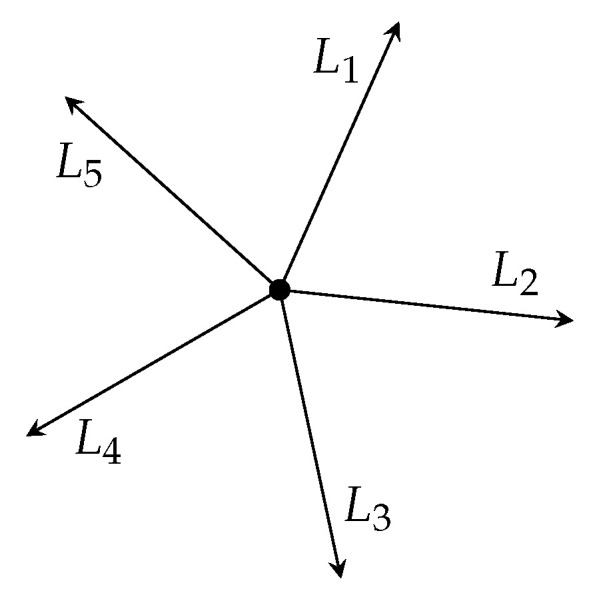
The graph of a diffusion spider with five legs.

If the angle is selected according to a discrete distribution, then there are at most countably many rays on which the diffusion lives. The state space of the process then corresponds to a star graph with edges of infinite length, and we call such a graph a spider. Thus, the Walsh Brownian motion can in this case be seen as an early example of a diffusion on a graph, and this process is called a Brownian spider. An example of a spider with five legs is given in Figure 1.

To make the diffusion more general, we can relax the requirement that the radial distance follows a Brownian motion and replace it with excursions from 0 of any regular reflected non-negative recurrent one-dimensional diffusion. In this paper, such a process is simply called a diffusion spider, which can also be seen as an abbreviation for “diffusion process on a spider”. The focus of this paper is on the occupation times on the legs of a diffusion spider, that is, the amount of time that the process is located on the different legs up to a given (fixed or random) time. If the underlying diffusion is not recurrent, we could in principle still study occupation times on the legs of the spider, but the problem loses much of its interest if the process at some point is located on a single leg without ever returning to the origin. For this reason, we only consider recurrent diffusions here.

Diffusions on graphs have been subjected to intensive research at least since the pioneering work by Freidlin and Wentzell [5]. We refer to Weber [6] for earlier references, but also for a study in the direction of our paper. In addition to [1,2,7] concerning diffusions on spiders, we recall, in particular, the papers by Papanicolaou, Papageorgiou, and Lepipas [8], Vakeroudis and Yor [9], Fitzsimmons and Kuter [10], Yano [4], Csáki, Csörgő, Földes and Révész [11,12], Ernst [13], Karatzas and Yan [14], Bayraktar and Zhang [15], Lempa, Mordecki and Salminen [16], and Bednarz, Ernst and Osękowski [17].

For results on occupation times and other earlier references, see [4] where the joint law of the occupation times on legs of a diffusion spider (there called a “multiray diffusion”) is analyzed via a double Laplace transform formula generalizing the results in [7] for a spider with excursions following a Bessel process. We also refer to [4] for a formula for the density of the joint law. Refs. [8,9] consider the occupation times for a Brownian spider, and [12] focus on limit theorems for local and occupation times for the Brownian spider. One could consider other stochastic processes on a spider as well, such as random walks [11,12] or continuous-time random walks. We leave the treatment of these interesting topics for eventual future work; however, in this paper, we focus on diffusion spiders, since the methods we apply for finding moment formulas do not lend themselves to a similar treatment of discrete or jump processes. A brief overview of the work by Révész et al. on random walks of spiders and Brownian spiders is given in [18].

As mentioned above, a skew diffusion can be seen as a special case of a diffusion spider. Namely, a one-dimensional diffusion which is skew at 0 corresponds to a spider with two legs (the positive and negative half-lines), which moves like its ordinary counterpart away from zero, but whenever it hits 0, it has a certain (skewed) probability of continuing to the positive side next. Due to this close relation, some applications of diffusion spiders can be anticipated by looking at applications of skew diffusions. The skew Brownian motion, in particular, has been used in models for a large number of phenomena, such as population dynamics over a boundary, ecosystems in rivers, pollutants diffusing in rock layers, shock acceleration of charged particles, and brain imaging; references are given by Lejay [19] and Ramirez et al. [20]. See Appuhamillage et al. [21] for results on the joint distribution of occupation and local times and applications in the dispersion of a solute concentration across an interface. Exact simulations of skew Brownian motion are discussed by Lejay and Pichot [22], statistical aspects by Lejay, Mordecki, and Torres [23], and applications in financial mathematics by Alvarez and Salminen [24], Rosello [25], and Hussain et al. [26]. Furthermore, we refer to Dassios and Zhang [27] for results on hitting times of Brownian spiders and, in particular, applications in the banking business. Finally, for an application of the Brownian spider in queueing theory, see Atar and Cohen [28].

This paper is structured as follows: In the next section, some key results from the theory of linear diffusions are presented, which are crucial in order to introduce and understand the notion of a diffusion spider briefly given in this section. We recall the explicit form of the Green function (resolvent density) derived in [16]. From this expression, we can immediately deduce some regularity properties of the Green function that are important in the subsequent analysis. The basic mathematical tool of the paper is an extension of Kac’s moment formula discussed in Section 3. The first main result, i.e., a recursive formula for the joint moments of the occupation times on the legs of a diffusion spider, is given in Theorem 3, Section 4. This can be seen as an extension of our previous results for one-dimensional diffusions in [29]. In Section 4, we also present a new formula for the joint Laplace transform of the occupation times, see Theorem 4, and connect this to earlier results by Barlow et al. [7] and Yano [4]. In Section 5, some examples are discussed, and we solve (see Theorem 5) the recursive equation for the joint moments for a Bessel spider—of which the Brownian spider is a special case. This is our third main result. At the end of Section 5, we also briefly return to the original Walsh Brownian motion. The proofs of the main results are given in the Appendix A, Appendix B, Appendix C, Appendix D and Appendix E at the end of the paper.

## 2. Preliminaries

### 2.1. Linear Diffusions

To make the paper more self-contained, we first recall the basic facts from the theory of linear diffusions needed to introduce the concept of a diffusion spider. Let $X={\left(X_{t}\right)}_{t\geq 0}$ be a linear diffusion living on $R_{+}=\left[0,+\infty \right)$. Let $P_{x}$ denote the probability measure associated with *X* when initiated at x≥0. For y≥0 introduce the first the hitting time via

$$H_{y}:=\inf\left\{t\geq 0:X_{t}=y\right\}.$$

It is assumed that *X* is regular and recurrent. Hence, for all x≥0 and y≥0 it holds that

$$P_{x}\left(H_{y}<\infty \right)=1.$$

Moreover, we suppose that 0 is a reflecting boundary and +∞ is a natural boundary (for the boundary classification for linear diffusions, see [30,31]). The $P_{x}$-distribution of $H_{y}$ is characterized for λ>0 via the Laplace transform

(1) $$E_{x}\left(e^{-\lambda H_{y}}\right)=\left\{\begin{array}{cc} \frac{\varphi_{\lambda }\left(x\right)}{\varphi_{\lambda }\left(y\right)}, & x\geq y,\\ \frac{\psi_{\lambda }\left(x\right)}{\psi_{\lambda }\left(y\right)}, & x\leq y, \end{array}\right.$$

where $E_{x}$ refers to the expectation operator associated with *X* and $\varphi_{\lambda }$ ($\psi_{\lambda }$) is a positive, continuous and decreasing (increasing) solution of the generalized differential equation

(2) $$Gu:=\frac{d}{dm}\frac{d}{dS}u=\lambda u,\;\lambda >0.$$

Here, *S* and *m* denote the scale function (strictly increasing and continuous) and the speed measure, respectively, associated with *X*. Under our assumptions, *m* is a positive measure. To fix ideas, we also assume that *m* does not have atoms and that $\varphi_{\lambda }$ and $\psi_{\lambda }$ are differentiable with respect to *S*. Recall that $\varphi_{\lambda }$ and $\psi_{\lambda }$ are unique solutions—up to multiplicative constants—of Equation (2) with the stated properties and satisfying the associated boundary conditions. Notice also that G, when operating in an appropriate function space, constitutes the infinitesimal generator of *X*. We also introduce the diffusion $X^{\partial }$ with the same speed and scale as *X* but for which 0 is a killing boundary. For $X^{\partial }$ there exist functions $\varphi_{\lambda }^{\partial }$ and $\psi_{\lambda }^{\partial }$ describing the distribution of $H_{y}$ for $X^{\partial }$ similarly as is conducted in (1) for *X*.

Recall that

(3) $$\psi^{\partial }\left(0\right)=0,\;\frac{d\psi }{dS}\left(0+\right)=0,\;\varphi^{\partial }\equiv \varphi ,$$

where the notation is shortened by omitting the subindex λ. Moreover, we normalize, as in [16],

(4) $$S\left(0\right)=0,\;\psi \left(0\right)=\varphi \left(0\right)=\varphi^{\partial }\left(0\right)=1,\;\mathrm{and}\;\frac{d\psi^{\partial }}{dS}\left(0+\right)=1.$$

As is well known, *X* has a transition density *p* with respect to *m*, i.e., for a Borel subset *A* of $R_{+}$,

$$P_{x}\left(X_{t}\in A\right)=\int_{A}p\left(t;x,y\right)\;m\left(dy\right),$$

and the Green function (resolvent density) is given by

(5) $$\begin{array}{cc} g_{\lambda }\left(x,y\right) & :=\int_{0}^{\infty }e^{-\lambda t}\;p\left(t;x,y\right)\;dt\\ \; & =\left\{\begin{array}{cc} w_{\lambda }^{-1}\psi \left(y\right)\varphi \left(x\right), & 0\leq y\leq x,\\ w_{\lambda }^{-1}\psi \left(x\right)\varphi \left(y\right), & 0\leq x\leq y, \end{array}\right. \end{array}$$

with the Wronskian

$$w_{\lambda }=\frac{d\psi }{dS}\left(x\right)\varphi \left(x\right)-\frac{d\varphi }{dS}\left(x\right)\psi \left(x\right)=-\frac{d\varphi }{dS}\left(0+\right).$$

For later use, recall that a diffusion *X* with starting point $X_{0}=0$ is called self-similar if for any a>0 there exists b>0 such that

$${\left(X_{at}\right)}_{t\geq 0}\overset{\left(d\right)}{=}{\left(bX_{t}\right)}_{t\geq 0}.$$

Perhaps the most well-known example of a self-similar diffusion is a standard Brownian motion starting in 0, for which the above identity holds with $b=\sqrt{a}$.

### 2.2. Diffusion Spider

Let $\Gamma \subset R^{2}$ be a star graph with one vertex at the origin of $R^{2}$ and *R* edges of infinite length meeting in the vertex (see Figure 1 for an example). Here, such a graph is called a spider. The edges $L_{1},\ldots ,L_{R}$ of the graph are known as the “rays” or—as hereafter called—the “legs” of the spider. The ordered pair (x,i) describes the point on Γ located on leg $L_{i}$ (i=1,…,R) at the distance x≥0 to the origin. We take the origin to be common to all legs, i.e.,

(0,1)=(0,2)=⋯=(0,R),

so for simplicity we just write 0 for the origin.

Let *X* be the linear diffusion introduced above. On the graph Γ we consider a stochastic process $X:={\left(X_{t}\right)}_{t\geq 0}$ using the notation

$$X_{t}:=\left(X_{t},\rho_{t}\right),$$

where $\rho_{t}\in \left\{1,2,\ldots ,R\right\}$ indicates the leg on which $X_{t}$ is located at time *t* and $X_{t}$ is the distance of $X_{t}$ to the origin at time *t* measured along the leg $L_{\rho_{t}}$. On each leg $L_{i}$, the process X behaves like the diffusion *X* until it hits 0. The process X is called a (homogeneous) diffusion spider. We could allow different diffusions on the different legs (the inhomogeneous case), but do not perform so in this paper. As part of the definition of the process, there are positive real numbers $\beta_{i}$, i=1,…,R, such that $\sum_{i=1}^{R}\beta_{i}=1$. When X hits 0, it continues, roughly speaking, with probability $\beta_{i}$ onto leg $L_{i}$. We do not here discuss the rigorous construction of the process, which can be performed, e.g., applying excursion theory; for this and other approaches, see the references given in the introduction. Notations $P_{\left(x,i\right)}$ and $E_{\left(x,i\right)}$ are used for the probability measure and expectation, respectively, when the diffusion spider starts at point (x,i), that is, on leg number *i* and at a distance *x* from the origin. As mentioned above, we write $P_{0}$ and $E_{0}$ without specifying a leg when the starting point is the origin.

For the diffusion spider X, we introduce its Green kernel (also called the resolvent kernel) via

$$G_{\lambda }u\left(x,i\right):=\int_{0}^{\infty }e^{-\lambda t}E_{\left(x,i\right)}\left(u(X_{t})\right)\;dt,$$

where (x,i)∈Γ, λ>0 and u:Γ→R is a bounded measurable function. Moreover, define

(6) $$\begin{array}{cc} \; & m\left(dx,i\right):=\beta_{i}m\left(dx\right),\;i=1,\ldots ,R,\;m\left(\left\{0\right\}\right):=0,\\ \; & S\left(dx,i\right):=\frac{1}{\beta_{i}}S\left(x\right). \end{array}$$

We call m and S the speed measure and the scale function, respectively, of X. Clearly, on every leg $L_{i}$ of the diffusion spider,

$$\frac{d}{dm}\frac{d}{dS}=\frac{d}{dm}\frac{d}{dS}.$$

Let $H_{0}=\inf\left\{t\geq 0:X_{t}=0\right\}$ be the first hitting time of 0 for the diffusion spider X. Since on every leg of the diffusion spider we have, loosely speaking, the same one-dimensional diffusion, for every i=1,2,…,R and x>0,

(7) $$E_{\left(x,i\right)}\left(e^{-\lambda H_{0}}\right)=E_{x}\left(e^{-\lambda H_{0}}\right)=\varphi_{\lambda }\left(x\right).$$

The following theorem, proved in [16], states an explicit expression for the resolvent density of X.

**Theorem 1.**  *The Green kernel of the diffusion spider X has a density $g_{\lambda }$ with respect to the speed measure m, which is given for x≥0 and y≥0 by*

(8) $$g_{\lambda }\left(\left(x,i\right),\left(y,j\right)\right)=\left\{\begin{array}{cc} \varphi \left(y\right)\tilde{\psi }\left(x,i\right), & \;x\leq y,\;i=j,\\ \varphi \left(x\right)\tilde{\psi }\left(y,i\right), & \;y\leq x,\;i=j,\\ c_{\lambda }^{-1}\;\varphi \left(y\right)\varphi \left(x\right), & \;i\neq j, \end{array}\right.$$

*where*

(9) $$\tilde{\psi }\left(x,i\right):=\frac{1}{\beta_{i}}\psi^{\partial }\left(x\right)+\frac{1}{c_{\lambda }}\varphi \left(x\right)$$

*and*

(10) $$c_{\lambda }:=-\frac{d}{dS}\varphi \left(0+\right)>0.$$

From the properties of the functions $\psi^{\partial }$ and φ, we immediately have the following result.

**Corollary 1.**  *The resolvent density (the Green function) $g_{\lambda }$ given in (8) is continuous on* Γ*, and for every i and j,*

(11) $$\lim_{(x,i)\to 0}g_{\lambda }\left(\left(x,i\right),\left(y,j\right)\right)=g_{\lambda }\left(0,\left(y,j\right)\right)=\frac{1}{c_{\lambda }}\varphi \left(y\right)=g_{\lambda }\left(0,y\right).$$

## 3. Kac’s Moment Formula

The tool that we will use to obtain the recursive expression for the joint moments is an extended variant of the Kac moment formula. Let *Y* be a regular diffusion taking values on an interval *E*. In spite of some conflict with our earlier notation, here we also let *m*, *p*, and $E_{x}$ (x∈E) denote the speed measure, the transition density and the expectation operator, respectively, associated with *Y*. Moreover, let V:E↦R be a measurable and bounded function and define for t>0 the additive functional

$$A_{t}\left(V\right):=\int_{0}^{t}V\left(Y_{s}\right)\;ds.$$

The moment formula by M. Kac for integral functionals, see [32], i.e.,

$$E_{x}\left({\left(A_{t}\left(V\right)\right)}^{n}\right)=n\int_{E}m\left(\;dy\right)\int_{0}^{t}p\left(s;x,y\right)V\left(y\right)E_{y}\left({\left(A_{t-s}\left(V\right)\right)}^{n-1}\right)\;ds,$$

is here extended into the following formula for the expected value of a product of powers of different functionals.

**Proposition 1.**  *Let $V_{1},\ldots ,V_{N}$ be measurable and bounded functions on E. For t>0, x∈E and $n_{1},\ldots ,n_{N}\in \left\{1,2,\ldots \right\}$,*

(12) $$E_{x}\left(\prod_{k=1}^{N}{\left(A_{t}\left(V_{k}\right)\right)}^{n_{k}}\right)=\sum_{k=1}^{N}n_{k}\int_{E}m\left(\;dy\right)\int_{0}^{t}p\left(s;x,y\right)V_{k}\left(y\right)E_{y}\left(\frac{\prod_{i=1}^{N}{\left(A_{t-s}\left(V_{i}\right)\right)}^{n_{i}}}{A_{t-s}\left(V_{k}\right)}\right)\;ds.$$

**Proof.**  See Appendix A.

The Formula in (12) is instrumental in the derivation of the results in Theorems 3 and 4 presented in Section 4.2.

## 4. Main Results

Let ${\left(X_{t}\right)}_{t\geq 0}$ be a diffusion spider with R≥2 legs meeting in the point 0, and let

$$A_{t}^{\left(i\right)}:=\int_{0}^{t}1_{L_{i}}\left(X_{s}\right)\;ds$$

be the occupation time on leg number *i* up to time *t*. Note that if the underlying diffusion *X* is self-similar, it follows for any *i* and any fixed t≥0 that

$$A_{t}^{\left(i\right)}\overset{\left(d\right)}{=}tA_{1}^{\left(i\right)},$$

meaning that for any such diffusion spider, we can equally well consider the occupation time up to time 1 instead of a general (fixed) time *t*.

**Figure 2:**
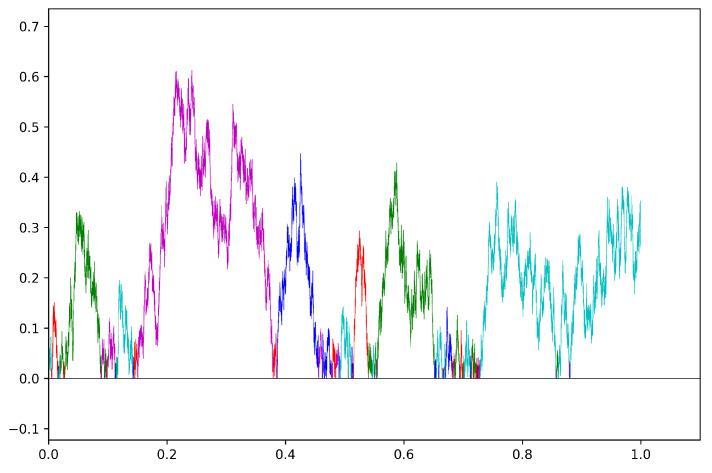
Sample path of the radial distance in a Brownian spider with five legs up to time 1. The five different colors indicate on which leg of the spider the process is located at any given time.

Figure 2 shows the radial distance from 0 as a function of time in a sample path of a Brownian spider with five legs. The excursions from 0 are colored to specify which leg the process is located on.

In this section we present formulas for recursively finding the moments of occupation times on the legs of a diffusion spider. In the first and shorter subsection we recapitulate the result for moments of a single occupation time, which is presented in our earlier paper [29]. In the second subsection this result is extended to joint moments of multiple occupation times.

### 4.1. Moments of the Occupation Time on a Single Leg

As pointed out in Section 6.4 of [29], the occupation time on a single leg $L_{i}$ of a (homogeneous) diffusion spider has the same law as the occupation time on the positive half-line of a one-dimensional skew diffusion process with the state space R and with the skewness parameter given by $\beta_{i}$. Namely, the spider is mapped onto R so that the leg $L_{i}$ corresponds to the positive half-line [0,∞), while all other legs are grouped together into a single second leg with parameter $\sum_{k\neq i}\beta_{k}=1-\beta_{i}$, which then is taken to be the negative half-line (−∞,0]. When considering the occupation time on the leg $L_{i}$; therefore, we can equally well consider the occupation time on [0,∞) of a one-dimensional diffusion.

The following results are shown in the previous paper [29], although here it is slightly modified to comply with the notation for diffusion spiders introduced in Section 2. In particular, note the differences mentioned in Remark 1.

**Theorem 2.**  *The Laplace transform of the first moment of $A_{t}^{\left(i\right)}$ is given by*

(13) $$L_{t}\left\{E_{0}\left(A_{t}^{\left(i\right)}\right)\right\}\left(\lambda \right)=\frac{1}{\lambda }\int_{0}^{\infty }g_{\lambda }\left(0,\left(y,i\right)\right)\beta_{i}\;m\left(\;dy\right)$$

*and for the higher moments, n≥2, recursively by*

(14) $$\begin{array}{cc} L_{t}\left\{E_{0}\left({\left(A_{t}^{\left(i\right)}\right)}^{n}\right)\right\}\left(\lambda \right)=\sum_{k=1}^{n} & \frac{n!D_{k}^{\left(i\right)}\left(\lambda \right)}{\left(n-k\right)!\lambda^{k}}L_{t}\left\{E_{0}\left({\left(A_{t}^{\left(i\right)}\right)}^{n-k}\right)\right\}\left(\lambda \right)\\ \; & +\frac{n!}{\lambda^{n-1}}L_{t}\left\{E_{0}\left(A_{t}^{\left(i\right)}\right)\right\}\left(\lambda \right)-\frac{n!}{\lambda^{n+1}}\sum_{k=1}^{n}D_{k}^{\left(j\right)}\left(\lambda \right), \end{array}$$

*where $L_{t}$ denotes the Laplace transform with respect to t of the function in curly brackets, λ is the Laplace parameter, and*

(15) $$D_{k}^{\left(i\right)}\left(\lambda \right):=\frac{\lambda^{k}}{(k-1)!}\int_{0}^{\infty }g_{\lambda }\left(0,\left(y,i\right)\right)E_{\left(y,i\right)}\left(H_{0}^{k-1}e^{-\lambda H_{0}}\right)\beta_{i}\;m\left(\;dy\right).$$

*Furthermore, if X is self-similar, then for any λ>0,*

(16) $$E_{0}\left({\left(A_{1}^{\left(i\right)}\right)}^{n}\right)=E_{0}\left(A_{1}^{\left(i\right)}\right)-\sum_{k=1}^{n}D_{k}^{\left(i\right)}\left(\lambda \right)\left(1-E_{0}\left({\left(A_{1}^{\left(i\right)}\right)}^{n-k}\right)\right),$$

*and, in particular, $D_{k}^{\left(i\right)}\left(\lambda \right)$ does not depend on λ for any i=1,…,R and k=1,2,….*

The proof is given in [29] (Theorem 2) with some minor notational differences.

**Remark 1.**  *Note the following:*

(1) *The one-dimensional diffusion X on R with speed measure m, in the setting of the other paper [29], here really corresponds to a two-legged diffusion spider X with speed measure m, which is why m(dx) has been replaced by $\beta_{i}m\left(dx\right)$ in (13) and (15), in accordance with (6).*
(2) *There is a sign change in the factor $D_{k}^{\left(i\right)}\left(\lambda \right)$ as defined in (15) compared to the corresponding expression in [29].*
(3) *The variable λ is not at all present on the left hand side of (16), and (using induction) we conclude that the factors $D_{k}^{\left(i\right)}\left(\lambda \right)$ cannot depend on λ either. Thus, the value λ>0 can be chosen arbitrarily. As a side note, this is the reason why a factor $\lambda^{k}$ is included in the expression for $D_{k}^{\left(i\right)}\left(\lambda \right)$ in (15).*

### 4.2. Joint Moments

The result in the previous section (from [29]) is here extended to a recursive formula for the Laplace transforms of the joint moments of the occupation times on multiple legs of a diffusion spider. For self-similar spiders we have a recursive formula directly for the joint moments, as in the case of the occupation time on one leg, cf. (16) in Theorem 2.

**Theorem 3.**  *For r∈{2,…,R} and $n_{1},\ldots ,n_{r}\geq 1$,*

(17) $$L_{t}\left\{E_{0}\left(\prod_{i=1}^{r}{\left(A_{t}^{\left(i\right)}\right)}^{n_{i}}\right)\right\}\left(\lambda \right)=\sum_{i=1}^{r}\sum_{k=1}^{n_{i}}\frac{n_{i}!D_{k}^{\left(i\right)}\left(\lambda \right)}{\left(n_{i}-k\right)!\lambda^{k}}L_{t}\left\{E_{0}\left(\frac{\prod_{j=1}^{r}{\left(A_{t}^{\left(j\right)}\right)}^{n_{j}}}{{\left(A_{t}^{\left(i\right)}\right)}^{k}}\right)\right\}\left(\lambda \right),$$

*where $D_{k}^{\left(i\right)}$ is defined in (15). If X is self-similar, then for any λ>0,*

(18) E0∏i=1r(A1(i))ni=∑i=1r∑k=1ninikn1+…+nrkDk(i)(λ)E0∏j=1r(A1(j))nj(A1(i))k.

**Proof.**  See Appendix B.

**Remark 2.**  *Despite the similarity, Theorem 2 does not follow by letting r=1 in Theorem 3. On the right-hand sides of equations (14) and (16) are not only the respective parts corresponding to (17) and (18) with r=1, but also some additional terms. This difference seems to originate from the fact that up to the first hitting time of 0, the occupation time on the starting leg is equal to the elapsed time (and, hence, positive), while the occupation time on any other leg is zero. Therefore, any product of occupation times on more than one leg is also zero up to time $H_{0}$, as can be seen in (A2), and even though we let the starting point tend to 0 when deriving the aforementioned theorems, there remains still a component which is nonzero in the case of a single leg but zero for the joint moments. With this in mind, Theorem 3 should not be seen as a replacement of Theorem 2 but as a complement to it.*

The result in Theorem 3 tells us that if we know the Green kernel of the diffusion spider X, we can recursively compute any joint moments of the occupation times on a number of legs. Recall also from (7) that for y>0

$$E_{\left(y,i\right)}\left(H_{0}^{k}e^{-\lambda H_{0}}\right)=E_{y}\left(H_{0}^{k}e^{-\lambda H_{0}}\right)={\left(-1\right)}^{k}\frac{\;d^{k}}{\;d\lambda^{k}}\varphi_{\lambda }\left(y\right),$$

i.e., we have all the ingredients needed to calculate the factors $D_{k}^{\left(i\right)}$ using the integral expression in (15).

The generalized version of Kac’s moment formula in Proposition 1 is now used to derive a moment generating function of the occupation times on the legs of a diffusion spider up to an exponential time *T*.

**Theorem 4.**  *Let T be exponentially distributed with mean 1/λ, λ>0, and independent of X. Then, for any $z_{1},\ldots ,z_{R}\geq 0$,*

(19) $$E_{0}\left(\exp\left(-\sum_{i=1}^{R}z_{i}A_{T}^{\left(i\right)}\right)\right)=\frac{1-\sum_{j=1}^{R}\frac{\lambda z_{j}}{\lambda +z_{j}}\int_{0}^{\infty }g_{\lambda }\left(0,\left(y,j\right)\right)\left(1-E_{\left(y,j\right)}\left(e^{-\left(\lambda +z_{j}\right)H_{0}}\right)\right)\beta_{j}\;m\left(\;dy\right)}{1+\sum_{j=1}^{R}z_{j}\int_{0}^{\infty }g_{\lambda }\left(0,\left(y,j\right)\right)E_{\left(y,j\right)}\left(e^{-\left(\lambda +z_{j}\right)H_{0}}\right)\beta_{j}\;m\left(\;dy\right)}.$$

**Proof.**  See Appendix C.

Formula (21) in the next corollary is due to Yano [4] (Theorem 3.5); see also Theorem 4 in Barlow, Pitman, and Yor [7], where the formula is presented for Bessel spiders (more on them in Section 5.1). We prove here that (20) is equivalent to our formula (19). For a Bessel spider, $c_{\lambda }$ is as given in (22), and notice that this formula shows that the inverse of the local time at 0 of the underlying reflecting Bessel process is a stable subordinator.

**Corollary 2.**  *For r∈{1,2,…,R} and $z_{i}>0,\;i=1,2,\ldots ,r,$*

(20) $$E_{0}\left(\exp\left(-\sum_{i=1}^{r}z_{i}A_{T}^{\left(i\right)}\right)\right)=\frac{1-\sum_{j=1}^{r}\beta_{j}+\sum_{j=1}^{r}\frac{\lambda \;\beta_{j}}{\lambda +z_{j}}\frac{c_{\lambda +z_{j}}}{c_{\lambda }}}{1-\sum_{j=1}^{r}\beta_{j}+\sum_{j=1}^{r}\beta_{j}\frac{c_{\lambda +z_{j}}}{c_{\lambda }}},$$

*where (cf. (10))*

$$c_{\lambda }:=-\frac{d}{dS}\varphi_{\lambda }\left(0+\right)>0.$$

*In particular, for $z_{i}>0$, i=1,2,…,R,*

(21) $$E_{0}\left(\exp\left(-\sum_{i=1}^{R}z_{i}A_{T}^{\left(i\right)}\right)\right)=\frac{1}{\sum_{j=1}^{R}\beta_{j}\;c_{\lambda +z_{j}}}\;\sum_{j=1}^{R}\frac{\lambda \;\beta_{j}\;c_{\lambda +z_{j}}}{\lambda +z_{j}}.$$

**Proof.**  See Appendix D.

## 5. Examples

In this section we highlight our results by analyzing a few different diffusion spiders, first and foremost Bessel spiders. For the Brownian spider, which is an important special case of Bessel spiders, it is possible to pursue the formulas further, and this evaluation is presented in a subsection of its own. Finally, we make some comments concerning occupation times for Walsh Brownian motion.

### 5.1. Bessel Spider

A Bessel process of dimension *n* and parameter ν:=n/2−1, where *n* is a positive integer, corresponds to the Euclidean norm of an *n*-dimensional Brownian motion. The *n*-dimensional Bessel process has the generator

$$Gf=\frac{1}{2}\frac{d^{2}f}{dx^{2}}+\frac{n-1}{2x}\frac{df}{dx},\;x>0,$$

which makes sense not only for integers *n* but any real values and, hence, any real parameter ν. We define the Bessel spider as a diffusion spider that behaves like a Bessel process with parameter ν∈(−1,0) (i.e., dimension 2+2ν) on each leg and has the corresponding excursion probabilities $\beta_{i}>0$, i=1,2,…R, such that $\beta_{1}+\ldots +\beta_{R}=1$. The restriction on ν to (−1,0) is so that the process is recurrent and hits 0; see [31] (p. 77). We now let X be a Bessel spider with R≥2 legs and apply the result in Theorem 3.

The Bessel spider has the self-similar property, which means that the recurrence equation in (18) applies. Recall that this recurrence hinges on the factors $D_{k}^{\left(i\right)}$ given in (15). For the purpose of finding $g_{\lambda }\left(0,\left(y,i\right)\right)$, that is, where the point *y* is on a particular leg $L_{i}$, we follow the procedure leading to Theorem 1. For the reflected Bessel diffusion on [0,+∞), we have from [31] (p. 137) that

$$m\left(dx\right)=2x^{2v+1}dx,\;S\left(x\right)=-\frac{1}{2\nu }x^{-2\nu },\;\varphi_{\lambda }\left(x\right)=x^{-\nu }K_{\nu }\left(x\sqrt{2\lambda }\right),$$

where $K_{\nu }$ is a modified Bessel function of the second kind. Then

(22) $$c_{\lambda }:=-\frac{d}{dS}\varphi \left(0+\right)={\left(\frac{2}{\sqrt{2\lambda }}\right)}^{\nu }\Gamma \left(\nu +1\right)=2^{\nu /2}\Gamma \left(\nu +1\right)\;\lambda^{-\nu /2}$$

and, hence,

$$g_{\lambda }\left(0,\left(y,i\right)\right)=\frac{1}{\Gamma (\nu +1)}{\left(\frac{\sqrt{2\lambda }}{2}\right)}^{\nu }y^{-\nu }K_{\nu }\left(y\sqrt{2\lambda }\right).$$

Note that $g_{\lambda }\left(0,\left(y,i\right)\right)$ is the same for all *i*. Similarly, the hitting time $H_{0}$ when starting in $y\in L_{i}$ corresponds precisely to the hitting time of zero in the reflected Bessel process. The values of $D_{k}^{\left(i\right)}$ are calculated as in [29] (Proof of Theorem 3), but note that a different normalization is used for *m* and *S* in that paper. This way, we get for any λ>0 that

(23) Dk(i)(λ)=−βiν+k−1k=−βik!∑j=1kkjνj,

where nk are unsigned Stirling numbers of the first kind. In the rest of this section, we will drop λ and only write $D_{k}^{\left(i\right)}$, as its value does not depend on λ.

As explained in Section 4.1, when only considering the occupation time on a single leg $L_{i}$, we can directly use the earlier obtained results for skew two-sided Bessel processes. Hence, by [29] (Theorem 4), the *n*th moment of the occupation time on $L_{i}$ up to time 1 is given by

(24) E0(A1(i))n=∑l=1n∑k=1l(−1)k−1Γ(k)Γ(n)nllkνl−1βik,

where nk are Stirling numbers of the second kind.

Using the recurrence equation in Theorem 3, the result in (24) is here extended to an explicit formula for the joint moments of the occupation times on multiple legs in a Bessel spider with R≥2 legs. With the numbering of legs being arbitrary, it should be clear that the formula—although written for the first *r* legs of the spider—holds when considering the occupation times on any number *r* of the *R* legs. Contrary to the recursive formula in Theorem 3 (see Remark 2), this formula also holds when r=1.

**Theorem 5.**  *For any r∈{1,…,R} and $n_{1},\ldots ,n_{r}\geq 1$,*

(25) E0∏i=1r(A1(i))ni=∑1≤k1≤l1≤n1⋯∑1≤kr≤lr≤nr(−1)K−1Γ(K)Γ(N)νL−1∏j=1rnjljljkjβjkj,

*where $N=n_{1}+\cdots +n_{r}$, $K=k_{1}+\cdots +k_{r}$ and $L=l_{1}+\ldots +l_{r}$.*

A proof of the theorem is given in Appendix E. For a particularly simple instance of the theorem above, consider the joint first moment of the occupation times on *r* legs in the Bessel spider.

**Corollary 3.**  *For any r∈{1,…,R},*

(26) $$E_{0}\left(A_{1}^{\left(1\right)}A_{1}^{\left(2\right)}\cdots A_{1}^{\left(r\right)}\right)={\left(-\nu \right)}^{r-1}\beta_{1}\beta_{2}\cdots \beta_{r}.$$

**Proof.**  Immediate from (25) with $n_{1}=\cdots =n_{r}=1$. □

The first few moments of the occupation times up to time *t* on one or two legs of a Bessel spider are given in Table 1. Recall that since the Bessel spider is self-similar, the (joint) moments of the occupation times on the legs up to a fixed time *t* satisfy

$$E_{0}\left({\left(A_{t}^{\left(i\right)}\right)}^{n_{1}}{\left(A_{t}^{\left(j\right)}\right)}^{n_{2}}\right)=t^{n_{1}+n_{2}}E_{0}\left({\left(A_{1}^{\left(i\right)}\right)}^{n_{1}}{\left(A_{1}^{\left(j\right)}\right)}^{n_{2}}\right).$$

Using this, the moments are found directly from Theorem 5. The variance, covariance, and correlation coefficients are also included in the table. Note, as expected, that the correlation coefficient is always negative and does not depend on the time *t* or the Bessel parameter ν. If the spider has only two legs, so that $\beta_{1}=1-\beta_{2}$, the process is always located on either of the legs and the correlation coefficient of the occupation times on the legs is then equal to −1.

### 5.2. Brownian Spider

The special case of a Bessel spider with the parameter $\nu =-\frac{1}{2}$ is the Brownian spider mentioned in the introduction, also known as Walsh Brownian motion on a finite number of legs. In this case, the result in Theorem 5 has the following, somewhat simpler expression.

**Theorem 6.**  *Let X be a Brownian spider and let $A_{1}^{\left(i\right)}$ be the occupation time on leg $L_{i}$ up to time 1. For any r∈{1,…,R} and $n_{1},\ldots ,n_{r}\geq 1$,*

(27) $$E_{0}\left(\prod_{i=1}^{r}{\left(A_{1}^{\left(i\right)}\right)}^{n_{i}}\right)=\sum_{k_{1}=1}^{n_{1}}\cdots \sum_{k_{r}=1}^{n_{r}}2^{-(2N-K-1)}\frac{\Gamma (K)}{\Gamma (N)}\prod_{j=1}^{r}\frac{\Gamma \left(2n_{j}-k_{j}\right)\;\beta_{j}^{k_{j}}}{\Gamma \left(k_{j}\right)\Gamma \left(n_{j}-k_{j}+1\right)},$$

*where $N=n_{1}+\cdots +n_{r}$ and $K=k_{1}+\cdots +k_{r}$.*

**Proof.**  The result follows from (25) and the identity

∑i=knniik(−2)n−i=(−1)n−k(2n−k−1)!2n−k(k−1)!(n−k)!=:b(n,k),

where b(n,k) is a (signed) Bessel number of the first kind. For proofs of this identity and some related ones, see [33,34]. □

### 5.3. Walsh Brownian Motion

Finally, we briefly return to the Walsh Brownian motion in its original form. As the state space can be the entire $R^{2}$ and is not restricted to a spider graph with a fixed number of legs, in this section we follow Walsh’s terminology and talk about “rays” rather than “legs”, although it should be clear that the meaning is the same. Here, the diffusion behaves like a Brownian motion on each ray, and when it reaches the origin, the direction θ of the next ray is selected according to some given distribution on [0,2π). As we have already studied the case when this distribution is discrete, i.e., the number of rays is at most countable, we now consider a continuous distribution.

**Figure 3:**
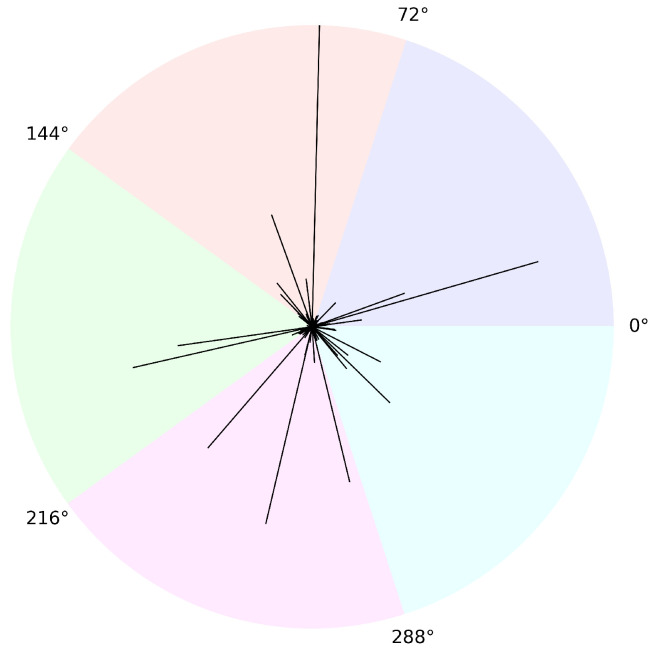
Plot of excursions from the origin of a simulated Walsh Brownian motion.

The diffusion will, almost surely, choose a new direction every time it reaches 0, so that it visits no ray more than once. Furthermore, the probability of visiting a particular ray (i.e., a ray whose angle is a fixed value) is zero. For this reason, it is not meaningful to consider the occupation times on specific rays in this case. Rather, we can consider the occupation times within sectors of the $R^{2}$ plane. Let $0=\theta_{0}<\theta_{1}<\theta_{2}<\cdots <\theta_{R}=2\pi$ be fixed angles and let $S_{i}$ consist of all points with angle in $\left[\theta_{i-1},\theta_{i}\right)$, so that $R^{2}$ is partitioned into *R* non-overlapping sectors $S_{1},S_{2},\ldots ,S_{R}$. If $\Theta (X_{t})$ denotes the angle of the ray on which the diffusion *X* is located at time *t*, then

$$A_{t}^{\left(S_{i}\right)}=\int_{0}^{t}1_{\left[\theta_{i-1},\theta_{i}\right)}\left(\Theta \left(X_{s}\right)\right)\;ds$$

is the occupation time of the diffusion within sector $S_{i}$ up to time *t*. This is illustrated in Figure 3, which contains a plot of a simulated Walsh Brownian motion. Each line corresponds to an excursion along a ray from the origin, and the length of each line is proportional to the maximal height of that excursion. The $R^{2}$ plane has been divided into five separate sectors of equal size, each with its own color, and we may consider the occupation time of the process in these sectors.

With respect to the occupation time on a sector, the outcome is the same as if all rays within the sector were combined and mapped onto a single ray. Therefore, the occupation times of a Walsh Brownian motion in the sectors $S_{1},\ldots ,S_{R}$ correspond precisely to the occupation times on the *R* legs of a Brownian spider. Thus, the result in Theorem 6 applies for the occupation times on sectors of a Walsh Brownian motion, with $\beta_{i}$ being equal to the probability of selecting an angle within sector $S_{i}$ when at the origin. Naturally, if the diffusion behaves like a Bessel process with parameter ν∈(−1,0) on each ray (this could, perhaps, be called a “Walsh Bessel process”), then Theorem 5 applies instead.

## Figures and Tables

**Table 1:** Some moments and descriptive statistics for the occupation times on the legs of a Bessel spider.

Moment/Statistic	Value
$E_{0}\left(A_{t}^{\left(i\right)}\right)$	$t\beta_{i}$
$E_{0}\left({\left(A_{t}^{\left(i\right)}\right)}^{2}\right)$	$t^{2}\beta_{i}\left(1+\nu -\nu \beta_{i}\right)$
$E_{0}\left(A_{t}^{\left(i\right)}A_{t}^{\left(j\right)}\right)$	$-t^{2}\nu \beta_{i}\beta_{j}$
$\mathrm{Var}\left(A_{t}^{\left(i\right)}\right)$	$t^{2}\left(1+\nu \right)\beta_{i}\left(1-\beta_{i}\right)$
$\mathrm{Cov}\left(A_{t}^{\left(i\right)},A_{t}^{\left(j\right)}\right),\;i\neq j$	$-t^{2}\left(1+\nu \right)\beta_{i}\beta_{j}$
$\mathrm{Corr}\left(A_{t}^{\left(i\right)},A_{t}^{\left(j\right)}\right),\;i\neq j$	$-\sqrt{\frac{\beta_{i}\beta_{j}}{\left(1-\beta_{i}\right)\left(1-\beta_{j}\right)}}$

## Data Availability

No new data were created or analyzed in this study. Data sharing is not applicable to this article.

## Appendix A

**Proof of Proposition 1.** We prove the statement for N=2, as the proof is analogous for any N>2. Restating the equation above with N=2, what we want to prove is that

$$\begin{array}{cc} E_{x}\left(A_{t}\left(V_{1}\right){{)}^{n_{1}}A_{t}\left(V_{2}\right))}^{n_{2}}\right)= & n_{1}\int_{E}m\left(\;dy\right)\int_{0}^{t}p\left(s;x,y\right)V_{1}\left(y\right)E_{y}\left(A_{t-s}\left(V_{1}\right){{)}^{n_{1}-1}A_{t-s}\left(V_{2}\right))}^{n_{2}}\right)\;ds\\ \; & +n_{2}\int_{E}m\left(\;dy\right)\int_{0}^{t}p\left(u;x,y\right)V_{2}\left(y\right)E_{y}\left(A_{t-u}\left(V_{1}\right){{)}^{n_{1}}A_{t-u}\left(V_{2}\right))}^{n_{2}-1}\right)\;du. \end{array}$$

Expanding the powers of $A_{t}\left(V_{1}\right)$ and $A_{t}\left(V_{2}\right)$ on the left hand side, we get

$$\begin{array}{cc} \; & E_{x}\left(A_{t}\left(V_{1}\right){{)}^{n_{1}}A_{t}\left(V_{2}\right))}^{n_{2}}\right)\\ \; & =E_{x}\left(\int_{0}^{t}\;ds_{1}\cdots \int_{0}^{t}\;ds_{n_{1}}\int_{0}^{t}\;du_{1}\cdots \int_{0}^{t}\;du_{n_{2}}\;V_{1}\left(Y_{s_{1}}\right)\cdots V_{1}\left(Y_{s_{n_{1}}}\right)\cdot V_{2}\left(Y_{u_{1}}\right)\cdots V_{2}\left(Y_{u_{n_{2}}}\right)\right)\\ \; & =n_{1}E_{x}\left(\int_{0}^{t}\;ds_{1}V_{1}\left(Y_{s_{1}}\right)\int_{s_{1}}^{t}\;ds_{2}\cdots \int_{s_{1}}^{t}\;du_{n_{2}}\;V_{1}\left(Y_{s_{2}}\right)\cdots V_{2}\left(Y_{u_{n_{2}}}\right)\right)\\ \; & \;+n_{2}E_{x}\left(\int_{0}^{t}\;du_{n_{2}}V_{2}\left(Y_{u_{n_{2}}}\right)\int_{u_{n_{2}}}^{t}\;ds_{1}\cdots \int_{u_{n_{2}}}^{t}\;du_{n_{2}-1}\;V_{1}\left(Y_{s_{1}}\right)\cdots V_{2}\left(Y_{u_{n_{2}-1}}\right)\right)\\ \; & =n_{1}\int_{0}^{t}\;ds_{1}E_{x}\left(V_{1}\left(Y_{s_{1}}\right)\int_{0}^{t-s_{1}}\;ds_{2}\cdots \int_{0}^{t-s_{1}}\;du_{n_{2}}\;V_{1}\left(Y_{s_{1}+s_{2}}\right)\cdots V_{2}\left(Y_{s_{1}+u_{n_{2}}}\right)\right)\\ \; & +n_{2}\int_{0}^{t}\;du_{n_{2}}E_{x}\left(V_{2}\left(Y_{u_{n_{2}}}\right)\int_{0}^{t-u_{n_{2}}}\;ds_{1}\cdots \int_{0}^{t-u_{n_{2}}}\;du_{n_{2}-1}\;V_{1}\left(Y_{u_{n_{2}}+s_{1}}\right)\cdots V_{2}\left(Y_{u_{n_{2}}+u_{n_{2}-1}}\right)\right)\\ \; & =n_{1}\int_{0}^{t}\;ds_{1}\int_{E}m\left(\;dy\right)p\left(s_{1};x,y\right)V_{1}\left(y\right)E_{y}\left(A_{t-s_{1}}\left(V_{1}\right){{)}^{n_{1}-1}A_{t-s_{1}}\left(V_{2}\right))}^{n_{2}}\right)\\ \; & \;+n_{2}\int_{0}^{t}\;du_{n_{2}}\int_{E}m\left(\;dy\right)p\left(u_{n_{2}};x,y\right)V_{2}\left(y\right)E_{y}\left(A_{t-u_{n_{2}}}\left(V_{1}\right){{)}^{n_{1}}A_{t-u_{n_{2}}}\left(V_{2}\right))}^{n_{2}-1}\right), \end{array}$$

where we have used the symmetry of the integrand in the second step and the strong Markov property in the final step. After a change of the order of the integration, justified by Fubini’s theorem, the desired result follows. □

**Remark A1.** 
*In the proof above, it is assumed that all values $n_{1},\ldots ,n_{N}$ are strictly positive integers. However, (12) may hold even if some (but not all) of these values are zero. Note that the factor $n_{k}$ in the terms on the right hand side ensures that any term with $n_{k}=0$ will not contribute, as long as the integral in that term is convergent. Suppose that for any starting point $Y_{0}=y$ of the underlying diffusion for which $V_{k}\left(y\right)\neq 0$, it almost surely holds that $A_{t}\left(V_{k}\right)>0$ when t>0, and $|A_{t}\left(V_{k}\right)\left|\geq \right|A_{t}\left(V_{i}\right)|,\forall i=1,\ldots ,N$ when t→0. Then, the denominator $A_{t-s}\left(V_{k}\right)$ inside the expected value is nonzero except possibly when s→t, in which case all factors in the numerator (and there is at least one) tend to zero as well, and at least as "fast". The first condition holds, for instance, if $V_{k}$ is non-negative everywhere and continuous in y, as the diffusion Y is assumed to be regular. Observe that one choice of functions $V_{k}$ that satisfies both these conditions is to take the indicator functions on the legs of a spider, i.e., $V_{k}=1_{L_{k}}$, which is the case of interest in this paper. Technically, the point 0 should be excluded from the indicator functions for the conditions to hold also when 0 is the starting point, but assuming that 0 is not a sticky point (as we perform in this paper), that will not make a difference.*

## Appendix B

**Proof of Theorem 3.** We first remark that the expression on the right hand side of (17) is well defined since $P_{0}\left(A_{t}^{\left(i\right)}>0\right)=1$ for all t>0 and i=1,2,…,R. The procedure below closely follows the proof of Theorem 2 in [29], with the main difference being that instead of the original Kac’s moment formula we use the generalized version given in Proposition 1. Proving the result for r=2 should be sufficient, since the method is the same for any higher values of *r* (you only need to include more terms of similar form). With r=2, the claimed identity (17) can be written as

(A1) $$\begin{array}{cc} \;L_{t}\left\{E_{0}\left({\left(A_{t}^{\left(1\right)}\right)}^{n_{1}}{\left(A_{t}^{\left(2\right)}\right)}^{n_{2}}\right)\right\}\left(\lambda \right) & =\sum_{k=1}^{n_{1}}\frac{n_{1}!D_{k}^{\left(1\right)}\left(\lambda \right)}{\left(n_{1}-k\right)!\lambda^{k}}L_{t}\left\{E_{0}\left({\left(A_{t}^{\left(1\right)}\right)}^{n_{1}-k}{\left(A_{t}^{\left(2\right)}\right)}^{n_{2}}\right)\right\}\left(\lambda \right)\\ \; & \;+\sum_{k=1}^{n_{2}}\frac{n_{2}!D_{k}^{\left(2\right)}\left(\lambda \right)}{\left(n_{2}-k\right)!\lambda^{k}}L_{t}\left\{E_{0}\left({\left(A_{t}^{\left(1\right)}\right)}^{n_{1}}{\left(A_{t}^{\left(2\right)}\right)}^{n_{2}-k}\right)\right\}\left(\lambda \right). \end{array}$$

Note that the numbering of the diffusion spider legs is arbitrary, so any two legs could be considered, even though they are here numbered 1 and 2. Assume first that the diffusion starts on one of the legs at a distance *x* from the origin. Without the loss of generality, we will write that it starts on the first leg $L_{1}$. Before the diffusion hits 0 for the first time, the occupation time on the starting leg $L_{1}$ equals the entire elapsed time, while the occupation time on any other leg stays zero. Thus, applying the strong Markov property at the first hitting time of 0, we have

$$A_{t}^{\left(1\right)}=\left\{\begin{array}{cc} H_{0}+A_{t-H_{0}}^{\left(1\right)}\circ \theta_{H_{0}}, & H_{0}<t,\\ t, & H_{0}\geq t, \end{array}\right.$$

while for any other leg,

$$A_{t}^{\left(k\right)}=\left\{\begin{array}{cc} A_{t-H_{0}}^{\left(k\right)}\circ \theta_{H_{0}}, & H_{0}<t,\\ 0, & H_{0}\geq t, \end{array}\right.\;\left(k>1\right).$$

where $\theta_{t}$ is the usual shift operator and the use of the composition ∘ should here be understood as

$$A_{t}^{\left(i\right)}\circ \theta_{u}:=\int_{0}^{t}1_{L_{i}}\left(\left(X_{s}\circ \theta_{u}\right)\left(\omega \right)\right)\;ds=\int_{0}^{t}1_{L_{i}}\left(X_{u+s}\right)\;ds.$$

From this we obtain, for any $n_{1},n_{2}\geq 1$,

(A2) (At(1))n1(At(2))n2=∑k=0n1n1kH0k(At−H0(1)∘θH0)n1−k(At−H0(2)∘θH0)n2,H0<t,0,H0≥t,

and

E(x,1)(At(1))n1(At(2))n2=∑k=0n1n1k∫0tE0(At−s(1))n1−k(At−s(2))n2skf(x,1;s)ds,

where f(x,1;t) denotes the $P_{\left(x,1\right)}$-density of $H_{0}$. Taking the Laplace transform with respect to *t*, we first recall that

$$L_{t}\left\{f\left(x,1;t\right)\right\}\left(\lambda \right)=\int_{0}^{\infty }e^{-\lambda t}P_{x}\left(H_{0}\in \;dt\right)=E_{\left(x,1\right)}\left(e^{-\lambda H_{0}}\right),$$

and

$$L_{t}\left\{t^{k}f\left(x,1;t\right)\right\}\left(\lambda \right)={\left(-1\right)}^{k}\frac{\;d^{k}}{\;d\lambda^{k}}L_{t}\left\{f\left(x,1;t\right)\right\}\left(\lambda \right)=E_{\left(x,1\right)}\left(H_{0}^{k}e^{-\lambda H_{0}}\right),$$

so that we obtain, using the formula for the Laplace transform of a convolution,

(A3) LtE(x,1)(At(1))n1(At(2))n2(λ)=∑k=0n1n1kE(x,1)H0ke−λH0LtE0(At(1))n1−k(At(2))n2(λ).

On the right hand side of the equation, the starting point (x,1) is only part of the expression involving the hitting time $H_{0}$, while the Laplace transform of the joint moments instead has the starting point 0. The equation above is now used together with Kac’s moment formula to obtain a recursive formula for (the Laplace transform of) the joint moments of the occupation times on the different legs. Proposition 1 yields

$$\begin{array}{cc} L_{t}\left\{E_{\left(x,1\right)}\left({\left(A_{t}^{\left(1\right)}\right)}^{n_{1}}{\left(A_{t}^{\left(2\right)}\right)}^{n_{2}}\right)\right\}\left(\lambda \right) & =n_{1}\int_{0}^{\infty }g_{\lambda }\left(\left(x,1\right),\left(y,1\right)\right)L_{t}\left\{E_{\left(y,1\right)}\left({\left(A_{t}^{\left(1\right)}\right)}^{n_{1}-1}{\left(A_{t}^{\left(2\right)}\right)}^{n_{2}}\right)\right\}\left(\lambda \right)\;\beta_{1}\;m\left(\;dy\right)\\ \; & +n_{2}\int_{0}^{\infty }g_{\lambda }\left(\left(x,1\right),\left(y,2\right)\right)L_{t}\left\{E_{\left(y,2\right)}\left({\left(A_{t}^{\left(1\right)}\right)}^{n_{1}}{\left(A_{t}^{\left(2\right)}\right)}^{n_{2}-1}\right)\right\}\left(\lambda \right)\;\beta_{2}\;m\left(\;dy\right), \end{array}$$

and inserting the expression in (A3) on both sides gives

∑k=0n1n1kE(x,1)H0ke−λH0LtE0(At(1))n1−k(At(2))n2(λ)=n1∑k=0n1−1n1−1kLtE0(At(1))n1−k−1(At(2))n2(λ)∫0∞gλ((x,1),(y,1))E(y,1)H0ke−λH0β1m(dy)+n2∑k=0n2−1n2−1kLtE0(At(1))n1(At(2))n2−k−1(λ)∫0∞gλ((x,1),(y,2))E(y,2)H0ke−λH0β2m(dy).

Note that the second integral is taken over points (y,2) that lie on the second leg $L_{2}$, which is why in that case the expression in (A3) is inserted with the roles of $n_{1}$ and $n_{2}$ interchanged. We now let x→0 on both sides of the equation above. For this, recall from [29] (Lemma 1) that

$$\lim_{x\to 0}E_{\left(x,1\right)}\left(H_{0}^{k}e^{-\lambda H_{0}}\right)=\left\{\begin{array}{cc} 1, & k=0,\\ 0, & k\geq 1, \end{array}\right.$$

and by Corollary 1 we may take the limit inside the integrals to obtain

$$\begin{array}{cc} \; & L_{t}\left\{E_{0}\left({\left(A_{t}^{\left(1\right)}\right)}^{n_{1}}{\left(A_{t}^{\left(2\right)}\right)}^{n_{2}}\right)\right\}\left(\lambda \right)\\ \; & \;=\sum_{k=1}^{n_{1}}\frac{n_{1}!}{(n_{1}-k)!}L_{t}\left\{E_{0}\left({\left(A_{t}^{\left(1\right)}\right)}^{n_{1}-k}{\left(A_{t}^{\left(2\right)}\right)}^{n_{2}}\right)\right\}\left(\lambda \right)\frac{1}{(k-1)!}\int_{0}^{\infty }g_{\lambda }\left(0,\left(y,1\right)\right)E_{\left(y,1\right)}\left(H_{0}^{k-1}e^{-\lambda H_{0}}\right)\;\beta_{1}\;m\left(\;dy\right)\\ \; & \;\;+\sum_{k=1}^{n_{2}}\frac{n_{2}!}{(n_{2}-k)!}L_{t}\left\{E_{0}\left({\left(A_{t}^{\left(1\right)}\right)}^{n_{1}}{\left(A_{t}^{\left(2\right)}\right)}^{n_{2}-k}\right)\right\}\left(\lambda \right)\frac{1}{(k-1)!}\int_{0}^{\infty }g_{\lambda }\left(0,\left(y,2\right)\right)E_{\left(y,2\right)}\left(H_{0}^{k-1}e^{-\lambda H_{0}}\right)\;\beta_{2}\;m\left(\;dy\right), \end{array}$$

where also the summation index is changed. This is equivalent to (A1) when introducing $D_{k}^{\left(i\right)}\left(\lambda \right)$ as defined in (15), and this proves the first part of the theorem. For a self-similar spider,

$${\left(A_{t}^{\left(1\right)}\right)}^{n_{1}}{\left(A_{t}^{\left(2\right)}\right)}^{n_{2}}\overset{\left(d\right)}{=}t^{n_{1}+n_{2}}{\left(A_{1}^{\left(1\right)}\right)}^{n_{1}}{\left(A_{1}^{\left(2\right)}\right)}^{n_{2}},$$

and, in this case,

$$L_{t}\left\{E_{0}\left({\left(A_{t}^{\left(1\right)}\right)}^{n_{1}}{\left(A_{t}^{\left(2\right)}\right)}^{n_{2}}\right)\right\}\left(\lambda \right)=\frac{(n_{1}+n_{2})!}{\lambda^{n_{1}+n_{2}+1}}E_{0}\left({\left(A_{1}^{\left(1\right)}\right)}^{n_{1}}{\left(A_{1}^{\left(2\right)}\right)}^{n_{2}}\right).$$

Hence, for self-similar spiders, the expression in (A1) simplifies to

(A4) E0(A1(1))n1(A1(2))n2=∑k=1n1n1kn1+n2kDk(1)(λ)E0(A1(1))n1−k(A1(2))n2+∑k=1n2n2kn1+n2kDk(2)(λ)E0(A1(1))n1(A1(2))n2−k.

This proves the second part of the theorem for r=2, and the proof is easily extended for higher *r*. □

## Appendix C

**Proof of Theorem 4.** If the diffusion spider starts in the point (x,j), that is, on a particular leg $L_{j}$, then up to the time $H_{0}$ the occupation time on the leg $L_{j}$ is equal to the time elapsed, while the occupation time on any other leg is zero. Hence, for any i∈{1,…,R},

$$A_{t}^{\left(i\right)}=\left\{\begin{array}{cc} H_{0}\;1_{\left\{i=j\right\}}+A_{t-H_{0}}^{\left(i\right)}\circ \theta_{H_{0}}, & H_{0}<t,\\ t\;1_{\left\{i=j\right\}}, & H_{0}\geq t. \end{array}\right.$$

To prove the theorem, we first consider the left hand side of (19) with a general starting point (x,j) instead of 0 and split the analysis into the two events whether the diffusion spider hits 0 before the exponential time *T* or not. This gives the two parts

$$\begin{array}{cc} E_{\left(x,j\right)}\left(\exp\left(-\sum_{i=1}^{R}z_{i}A_{T}^{\left(i\right)}\right);H_{0}\geq T\right)\; & =E_{\left(x,j\right)}\left(e^{-z_{j}T};H_{0}\geq T\right)\\ \; & =\frac{\lambda }{\lambda +z_{j}}\left(1-E_{\left(x,j\right)}\left(e^{-\left(\lambda +z_{j}\right)H_{0}}\right)\right) \end{array}$$

and

$$\begin{array}{cc} E_{\left(x,j\right)}\left(\exp\left(-\sum_{i=1}^{R}z_{i}A_{T}^{\left(i\right)}\right);H_{0}<T\right)\; & =E_{\left(x,j\right)}\left(e^{-z_{j}H_{0}};H_{0}<T\right)E_{0}\left(\exp\left(-\sum_{i=1}^{R}z_{i}A_{T}^{\left(i\right)}\right)\right)\\ \; & =E_{\left(x,j\right)}\left(e^{-\left(\lambda +z_{j}\right)H_{0}}\right)E_{0}\left(\exp\left(-\sum_{i=1}^{R}z_{i}A_{T}^{\left(i\right)}\right)\right), \end{array}$$

where in the second part we have used the strong Markov property to restart the process when it first hits 0, as well as the memoryless property of the exponential distribution. Combining both parts gives

(A5) $$E_{\left(x,j\right)}\left(\exp\left(-\sum_{i=1}^{R}z_{i}A_{T}^{\left(i\right)}\right)\right)=\frac{\lambda }{\lambda +z_{j}}+\left(E_{0}\left(\exp\left(-\sum_{i=1}^{R}z_{i}A_{T}^{\left(i\right)}\right)\right)-\frac{\lambda }{\lambda +z_{j}}\right)E_{\left(x,j\right)}\left(e^{-\left(\lambda +z_{j}\right)H_{0}}\right).$$

The significance of this expression is that on the right hand side the dependency on the starting position (x,j) is contained only in a function of the first hitting time $H_{0}$, while the moment generating function of the occupation times has the starting point 0 instead. As the left hand side of (A5) can be written

(A6) $$E_{\left(x,j\right)}\left(\exp\left(-\sum_{i=1}^{R}z_{i}A_{T}^{\left(i\right)}\right)\right)=\int_{0}^{\infty }E_{\left(x,j\right)}\left(\exp\left(-\sum_{i=1}^{R}z_{i}A_{t}^{\left(i\right)}\right)\right)\lambda e^{-\lambda t}\;dt,$$

we will, for the moment, consider the expression with a fixed time *t* rather than the exponential time *T*. Expanding as a sum of joint moments, we obtain

(A7) $$\begin{array}{cc} E_{\left(x,j\right)}\left(\exp\left(-\sum_{i=1}^{R}z_{i}A_{t}^{\left(i\right)}\right)\right)\; & =\sum_{N=0}^{\infty }\frac{{\left(-1\right)}^{N}}{N!}E_{\left(x,j\right)}\left({\left(\sum_{i=1}^{R}z_{i}A_{t}^{\left(i\right)}\right)}^{N}\right)\\ \; & =\sum_{N=0}^{\infty }\frac{{\left(-1\right)}^{N}}{N!}E_{\left(x,j\right)}\left(\sum_{\begin{array}{c} n_{1},\ldots ,n_{R}\geq 0\\ n_{1}+\cdots +n_{R}=N \end{array}}\;\frac{N!}{n_{1}!n_{2}!\cdots n_{R}!}\prod_{i=1}^{R}{\left(z_{i}A_{t}^{\left(i\right)}\right)}^{n_{i}}\right)\\ \; & =\sum_{N=0}^{\infty }\sum_{\begin{array}{c} n_{1},\ldots ,n_{R}\geq 0\\ n_{1}+\cdots +n_{R}=N \end{array}}\left(\prod_{l=1}^{R}\frac{{\left(-z_{l}\right)}^{n_{l}}}{n_{l}!}\right)E_{\left(x,j\right)}\left(\prod_{i=1}^{R}{\left(A_{t}^{\left(i\right)}\right)}^{n_{i}}\right). \end{array}$$

The generalized Kac’s moment Formula (12) with the functions $V_{k}\left(x\right)=1_{L_{k}}\left(x\right)$ (and formulated for the spider) becomes

(A8) $$E_{\left(x,j\right)}\left(\prod_{i=1}^{R}{\left(A_{t}^{\left(i\right)}\right)}^{n_{i}}\right)=\sum_{i=1}^{R}n_{i}\int_{0}^{\infty }\beta_{i}m\left(\;dy\right)\int_{0}^{t}p\left(s;\left(x,j\right),\left(y,i\right)\right)E_{\left(y,i\right)}\left(\frac{\prod_{k=1}^{R}{\left(A_{t-s}^{\left(k\right)}\right)}^{n_{k}}}{A_{t-s}^{\left(i\right)}}\right)\;ds,$$

where p(s;(x,j),(y,i)) denotes the transition density of the spider. Next, Equation (A8) is inserted in (A7), bearing in mind that by Remark A1 this can be performed even when some of the values $n_{1},\ldots ,n_{R}$ are zero. The exception is the term for N=0, since at least one of the $n_{i}$ has to be strictly positive, so this term (which evaluates to 1) is separated from the rest of the sum. This yields

$$\begin{array}{cc} \; & E_{\left(x,j\right)}\left(\exp\left(-\sum_{i=1}^{R}z_{i}A_{t}^{\left(i\right)}\right)\right)\\ \; & \;=1+\sum_{N=1}^{\infty }\sum_{\begin{array}{c} n_{1},\ldots ,n_{R}\geq 0\\ n_{1}+\cdots +n_{R}=N \end{array}}\left(\prod_{l=1}^{R}\frac{{\left(-z_{l}\right)}^{n_{l}}}{n_{l}!}\right)\sum_{i=1}^{R}n_{i}\int_{0}^{\infty }\beta_{i}\;m\left(\;dy\right)\int_{0}^{t}p\left(s;\left(x,j\right),\left(y,i\right)\right)E_{\left(y,i\right)}\left(\frac{\prod_{k=1}^{R}{\left(A_{t-s}^{\left(k\right)}\right)}^{n_{k}}}{A_{t-s}^{\left(i\right)}}\right)\;ds\\ \; & \;=1+\sum_{i=1}^{R}\int_{0}^{\infty }\beta_{i}\;m\left(\;dy\right)\int_{0}^{t}p\left(s;\left(x,j\right),\left(y,i\right)\right)\sum_{N=0}^{\infty }\sum_{\begin{array}{c} n_{1},\ldots ,n_{R}\geq 0\\ n_{1}+\cdots +n_{R}=N \end{array}}\left(-z_{i}\right)\left(\prod_{l=1}^{R}\frac{{\left(-z_{l}\right)}^{n_{l}}}{n_{l}!}\right)E_{\left(y,i\right)}\left(\prod_{k=1}^{R}{\left(A_{t-s}^{\left(k\right)}\right)}^{n_{k}}\right)\;ds\\ \; & \;=1-\sum_{i=1}^{R}z_{i}\int_{0}^{\infty }\beta_{i}\;m\left(\;dy\right)\int_{0}^{t}p\left(s;\left(x,j\right),\left(y,i\right)\right)E_{\left(y,i\right)}\left(\exp\left(-\sum_{i=1}^{R}z_{i}A_{t-s}^{\left(i\right)}\right)\right)\;ds. \end{array}$$

Note that the summation indices *N* and $n_{i}$ have both been shifted by one in the third step and that in the last step (A7) has been applied again. From (A6) and the above, we obtain

(A9) $$\begin{array}{cc} \; & E_{\left(x,j\right)}\left(\exp\left(-\sum_{i=1}^{R}z_{i}A_{T}^{\left(i\right)}\right)\right)=\lambda \;L_{t}\left\{E_{\left(x,j\right)}\left(\exp\left(-\sum_{i=1}^{R}z_{i}A_{t}^{\left(i\right)}\right)\right)\right\}\left(\lambda \right)\\ \; & =\frac{\lambda }{\lambda }-\lambda \sum_{i=1}^{R}z_{i}\int_{0}^{\infty }L_{t}\left\{\int_{0}^{t}p\left(s;\left(x,j\right),\left(y,i\right)\right)E_{\left(y,i\right)}\left(\exp\left(-\sum_{i=1}^{R}z_{i}A_{t-s}^{\left(i\right)}\right)\right)\;ds\right\}\left(\lambda \right)\;\beta_{i}\;m\left(\;dy\right)\\ \; & =1-\lambda \sum_{i=1}^{R}z_{i}\int_{0}^{\infty }g_{\lambda }\left(\left(x,j\right),\left(y,i\right)\right)L_{t}\left\{E_{\left(y,i\right)}\left(\exp\left(-\sum_{i=1}^{R}z_{i}A_{t}^{\left(i\right)}\right)\right)\right\}\left(\lambda \right)\;\beta_{i}\;m\left(\;dy\right)\\ \; & =1-\sum_{i=1}^{R}z_{i}\int_{0}^{\infty }g_{\lambda }\left(\left(x,j\right),\left(y,i\right)\right)E_{\left(y,i\right)}\left(\exp\left(-\sum_{i=1}^{R}z_{i}A_{T}^{\left(i\right)}\right)\right)\beta_{i}\;m\left(\;dy\right). \end{array}$$

We now insert (A5) into the right hand side of (A9) and let x→0 on both sides. This gives

$$\begin{array}{cc} E_{0}\left(\exp\left(-\sum_{i=1}^{R}z_{i}A_{T}^{\left(i\right)}\right)\right) & =1-\sum_{i=1}^{R}z_{i}\int_{0}^{\infty }g_{\lambda }\left(0,\left(y,i\right)\right)\frac{\lambda }{\lambda +z_{i}}\left(1-E_{\left(y,i\right)}\left(e^{-\left(\lambda +z_{i}\right)H_{0}}\right)\right)\beta_{i}\;m\left(\;dy\right)\\ & \;-E_{0}\left(\exp\left(-\sum_{i=1}^{R}z_{i}A_{T}^{\left(i\right)}\right)\right)\sum_{i=1}^{R}z_{i}\int_{0}^{\infty }g_{\lambda }\left(0,\left(y,i\right)\right)E_{\left(y,i\right)}\left(e^{-\left(\lambda +z_{i}\right)H_{0}}\right)\beta_{i}\;m\left(\;dy\right), \end{array}$$

which, when solved for the left hand side expression, results in the claimed formula (19). □

## Appendix D

**Proof of Corollary 2.** Recall from (11) that

$$g_{\lambda }\left(0,\left(y,j\right)\right)=\frac{1}{c_{\lambda }}\;\varphi_{\lambda }\left(y\right)$$

and from (7)

$$E_{\left(y,j\right)}\left(e^{-\left(\lambda +z_{j}\right)H_{0}}\right)=\varphi_{\lambda +z_{j}}\left(y\right).$$

Hence, (19) can be rewritten

$$E_{0}\left(\exp\left(-\sum_{i=1}^{r}z_{i}A_{T}^{\left(i\right)}\right)\right)=\frac{1-\sum_{j=1}^{r}\frac{\lambda \;\beta_{j}\;z_{j}}{c_{\lambda }\;\left(\lambda +z_{j}\right)}\left(\int_{0}^{\infty }\varphi_{\lambda }\left(y\right)\;m\left(\;dy\right)-\int_{0}^{\infty }\varphi_{\lambda }\left(y\right)\varphi_{\lambda +z_{j}}\left(y\right)\;m\left(\;dy\right)\right)}{1+\sum_{j=1}^{r}\frac{\beta_{j}\;z_{j}}{c_{\lambda }}\int_{0}^{\infty }\varphi_{\lambda }\left(y\right)\varphi_{\lambda +z_{j}}\left(y\right)\;m\left(\;dy\right)}.$$

For the integrals, we have (cf. [29], proof of Corollary 1)

$$\begin{array}{cc} \lambda \int_{0}^{\infty }\varphi_{\lambda }\left(y\right)\;m\left(\;dy\right) & =c_{\lambda },\\ z_{j}\int_{0}^{\infty }\varphi_{\lambda }\left(y\right)\varphi_{\lambda +z_{j}}\left(y\right)\;m\left(\;dy\right) & =-c_{\lambda }+c_{\lambda +z_{j}} \end{array}$$

by which the previous equation becomes

$$E_{0}\left(\exp\left(-\sum_{i=1}^{r}z_{i}A_{T}^{\left(i\right)}\right)\right)=\frac{1-\sum_{j=1}^{r}\left(\frac{\lambda \;\beta_{j}\;z_{j}}{\lambda +z_{j}}\right)\left(\frac{1}{\lambda }+\frac{1}{z_{j}}\right)+\sum_{j=1}^{r}\frac{\lambda \;\beta_{j}}{\lambda +z_{j}}\frac{c_{\lambda +z_{j}}}{c_{\lambda }}}{1-\sum_{j=1}^{r}\beta_{j}+\sum_{j=1}^{r}\beta_{j}\frac{c_{\lambda +z_{j}}}{c_{\lambda }}}$$

from which (20) easily follows, and (21) is immediate since $\sum_{j=1}^{R}\beta_{j}=1.$ □

## Appendix E

**Proof of Theorem 5.** The known Equation (24) coincides with (25) where r=1, showing that the theorem holds in that particular case. As in the proof of Theorem 3, we here prove the statement for r=2, say the two legs $L_{1}$ and $L_{2}$ in the Bessel spider. This will be enough to demonstrate the procedure, which can then readily be repeated for a larger value of *r* with more tedious but hardly more difficult work. To begin with, we repeat the statement in (25) for r=2:

(A10) E0(A1(1))n1(A1(2))n2=∑l1=1n1∑k1=1l1∑l2=1n2∑k2=1l2(−1)k1+k2−1Γ(k1+k2)Γ(n1+n2)n1l1l1k1n2l2l2k2νl1+l2−1β1k1β2k2.

We prove this statement by induction using the recurrence in (18) and the known moment formula (24) for the occupation time on a single leg. For the simplest case $n_{1}=n_{2}=1$, we see from (18) that

$$E_{0}\left(A_{1}^{\left(1\right)}A_{1}^{\left(2\right)}\right)=\frac{1}{2}D_{1}^{\left(1\right)}E_{0}\left(A_{1}^{\left(2\right)}\right)+\frac{1}{2}D_{1}^{\left(2\right)}E_{0}\left(A_{1}^{\left(1\right)}\right)=-\beta_{1}\beta_{2}\nu ,$$

since $D_{1}^{\left(i\right)}=-\beta_{i}\nu$ and $E_{0}\left(A_{1}^{\left(i\right)}\right)=\beta_{i}$. Thus, (A10) holds in this case. Assume now that (A10) holds whenever $\left\{1\leq n_{1}\leq a-1,1\leq n_{2}\leq b\right\}$ or $\left\{1\leq n_{1}\leq a,1\leq n_{2}\leq b-1\right\}$ for some integers a,b≥1. We proceed to show that then (A10) holds also for $n_{1}=a,n_{2}=b$. First, we apply the recurrence Equation (18) to obtain

(A11) E0(A1(1))a(A1(2))b=Da(1)a+baE0(A1(2))b+∑i=1a−1aia+biDi(1)E0(A1(1))a−i(A1(2))b+Db(2)a+bbE0(A1(1))a+∑i=1b−1bia+biDi(2)E0(A1(1))a(A1(2))b−i.

Here, we have separated the terms with moments of the occupation time on only one leg, for which (24) applies, and the terms with joint moments of the occupation times on both legs, for which we can apply the induction assumption. In the first case, we insert the expressions in (23) and (24) to obtain

(A12) Da(1)a+baE0(A1(2))b=1a+ba−β1a!∑l1=1aal1νl1∑l2=1b∑k2=1l2(−1)k2−1Γ(k2)Γ(b)bl2l2k2νl2−1β2k2=b∑l1=1a∑k1=11∑l2=1b∑k2=1l2(−1)k1+k2−1Γ(k1+k2−1)Γ(a+b+1)al1l1k1bl2l2k2νl1+l2−1β1k1β2k2.

Note that the variable $k_{1}$ only takes the value 1 here, but it is nevertheless added so that the expression above resembles the form of (A10) more closely. Next, we turn to the following term in (A11), assuming for the moment that a>1 so that the sum is not empty. Inserting (23) and the induction assumption, we obtain

(A13) ∑i=1a−1aia+biDi(1)E0(A1(1))a−i(A1(2))b=∑i=1a−1aia+bb+iDa−i(1)E0(A1(1))i(A1(2))b=∑i=1a−1aia+bb+i−β1(a−i)!∑j=1a−ia−ijνj∑1≤k1≤l1≤i,1≤k2≤l2≤b(−1)k1+k2−1Γ(k1+k2)Γ(b+i)il1bl2l1k1l2k2νl1+l2−1β1k1β2k2=∑1≤k1≤l1≤i≤j≤a−1,1≤k2≤l2≤b(−1)k1+k2νa+l1+l2−j−1β1k1+1β2k2(b+i)Γ(k1+k2)Γ(a+b+1)aiil1bl2l1k1l2k2a−ia−j=∑1≤k1≤j≤a−1,1≤k2≤l2≤b(−1)k1+k2νl2+jβ1k1+1β2k2Γ(k1+k2)Γ(a+b+1)bl2l2k2∑l1=k1jl1k1∑i=l1a+l1−j−1(b+i)aiil1a−ij−l1+1,

where, in the last step, we have changed the order of summation according to the pattern

$$\begin{array}{cc} \sum_{k=1}^{n}\sum_{l=k}^{n}\sum_{i=l}^{n}\sum_{j=i}^{n}f\left(i,j,k,l\right)\; & =\sum_{k=1}^{n}\sum_{l=k}^{n}\sum_{i=l}^{n}\sum_{j=l}^{n+l-i}f\left(i,n+l-j,k,l\right)\\ \; & =\sum_{k=1}^{n}\sum_{j=k}^{n}\sum_{l=k}^{j}\sum_{i=l}^{n+l-j}f\left(i,n+l-j,k,l\right). \end{array}$$

To simplify the expression further, we use the two closely related identities

∑i=kn−mikn−imni=k+mknk+m

and

∑i=kn−mi+1k+1n−imni=k+mkn+1k+m+1,

the first of which is well known [35] and is also utilized in the proof of the latter (see Lemma 2 in [29]). Applying these identities, we know that the innermost sum in (A13) is equal to

∑i=l1a+l1−j−1(b+i)aiil1a−ij−l1+1=b∑i=l1a−(j−l1+1)aiil1a−ij−l1+1+a∑i=l1a−(j−l1+1)a−1i−1il1a−ij−l1+1=bj+1l1aj+1+ajl1−1aj+1=aj+1bjl1+(a+b)jl1−1.

The expression inside the parenthesis in (A13) becomes

∑l1=k1jl1k1∑i=l1a+l1−j−1(b+i)aiil1a−ij−l1+1=aj+1b∑l1=k1jl1k1jl1+(a+b)∑l1=k1jl1k1jl1−1=aj+1j+1k1+1b+(a+b)k1,

where the second step follows by the identities

∑i=knikni=n+1k+1,∑i=knikni−1=kn+1k+1,

see Equation (6.15) in [35] and the proof of Theorem 4 in [29], respectively. Inserting this into (A13) gives

∑i=1a−1aia+biDi(1)E0(A1(1))a−i(A1(2))b=∑1≤k1≤j≤a−1,1≤k2≤l2≤b(−1)k1+k2νl2+jβ1k1+1β2k2Γ(k1+k2)Γ(a+b+1)bl2l2k2aj+1j+1k1+1b+(a+b)k1=∑2≤k1≤j≤a,1≤k2≤l2≤b(−1)k1+k2−1νl2+j−1β1k1β2k2Γ(k1+k2−1)Γ(a+b+1)ajjk1bl2l2k2b+(a+b)(k1−1).

When this is combined with (A12), replacing the summation index *j* with $l_{1}$ in the process, the result is

Da(1)a+baE0(A1(2))b+∑i=1a−1aia+biDi(1)E0(A1(1))a−i(A1(2))b=∑1≤k1≤l1≤a,1≤k2≤l2≤b(−1)k1+k2−1Γ(k1+k2−1)Γ(a+b+1)al1l1k1bl2l2k2νl1+l2−1β1k1β2k2b+(a+b)(k1−1).

Note that this coincides with (A12) when a=1, so the temporary assumption a>1 is not necessary for this expression to hold. This is half the right hand side of (A11). We obtain the other half simply by interchanging the roles of *a* and *b* in the expression above (renaming the summation indices accordingly). Thus, adding all the terms together, we get

E0(A1(1))a(A1(2))b=∑1≤k1≤l1≤a,1≤k2≤l2≤b(−1)k1+k2−1Γ(k1+k2−1)Γ(a+b+1)al1l1k1bl2l2k2νl1+l2−1β1k1β2k2·b+(a+b)(k1−1)+a+(a+b)(k2−1)=∑1≤k1≤l1≤a,1≤k2≤l2≤b(−1)k1+k2−1Γ(k1+k2)Γ(a+b)al1l1k1bl2l2k2νl1+l2−1β1k1β2k2,

which proves that (A10) indeed holds for $n_{1}=a,n_{2}=b$. By induction, it holds for any $n_{1},n_{2}\geq 1$, and the theorem is thereby proved when r=2. As mentioned in the beginning of the proof, the same method can be repeated for increasingly larger values of *r* as well. However, due to the long expressions we include only the proof given above and trust that the reader can recognize how it generalizes to (25) for higher *r*. □

## References

[1] Walsh J.B. (1978). A diffusion with a discontinuous local time. Temps locaux.

[2] Barlow M., Pitman J., Yor M. (1989). On Walsh’s Brownian motions. Séminaire de Probabilités, XXIII.

[3] Salisbury T.S. (1986). Construction of right processes from excursions. Probab. Theory Related Fields.

[4] Yano Y. (2017). On the joint law of the occupation times for a diffusion process on multiray. J. Theoret. Probab..

[5] Freidlin M.I., Wentzell A.D. (1993). Diffusion processes on graphs and the averaging principle. Ann. Probab..

[6] Weber M. (2001). On occupation time functionals for diffusion processes and birth-and-death processes on graphs. Ann. Appl. Probab..

[7] Barlow M., Pitman J., Yor M. (1989). Une extension multidimensionnelle de la loi de l’arc sinus. Séminaire de Probabilités, XXIII.

[8] Papanicolaou V., Papageorgiou E., Lepipas D. (2012). Random motion on simple graphs. Methodol. Comput. Appl. Probab..

[9] Vakeroudis S., Yor M. (2012). A scaling proof for Walsh’s Brownian motion extended arc-sine law. Electron. Commun. Probab..

[10] Fitzsimmons P.J., Kuter K.E. (2014). Harmonic functions on Walsh’s Brownian motion. Stoch. Process. Appl..

[11] Csáki E., Csörgő M., Földes A., Révész P. (2016). Some limit theorems for heights of random walks on a spider. J. Theoret. Probab..

[12] Csáki E., Csörgő M., Földes A., Révész P. (2019). Limit theorems for local and occupation times of random walks and Brownian motion on a spider. J. Theoret. Probab..

[13] Ernst P. (2016). Exercising control when confronted by a (Brownian) spider. Oper. Res. Lett..

[14] Karatzas I., Yan M. (2019). Semimartingales on rays, Walsh diffusions, and related problems of control and stopping. Stoch. Process. Appl..

[15] Bayraktar E., Zhang X. (2021). Embedding of Walsh Brownian motion. Stoch. Process. Appl..

[16] Lempa J., Mordecki E., Salminen P. (2024). Diffusion spiders: Green kernel, excessive functions and optimal stopping. Stoch. Process. Appl..

[17] Bednarz E., Ernst P.A., Osękowski A. (2024). On the diameter of the stopped spider process. Math. Oper. Res..

[18] Csáki E., Földes A. (2024). In memoriam Pál Révész (1934–2022). Period. Math. Hungar..

[19] Lejay A. (2006). On the constructions of the skew Brownian motion. Probab. Surv..

[20] Ramirez J.M., Thomann E.A., Waymire E.C. (2013). Advection-dispersion across interfaces. Statist. Sci..

[21] Appuhamillage T., Bokil V., Thomann E., Waymire E., Wood B. (2011). Occupation and local times for skew Brownian motion with applications to dispersion across an interface. Ann. Appl. Probab..

[22] Lejay A., Pichot G. (2012). Simulating diffusion processes in discontinuous media: Numerical scheme with constant time step. J. Comput. Phys..

[23] Lejay A., Mordecki E., Torres S. (2014). Is a Brownian motion skew?. Scand. J. Statist..

[24] Alvarez L., Salminen P. (2017). Timing in the presence of directional predictability: Optimal stopping of skew Brownian motion. Math. Meth. Oper. Res..

[25] Rossello D. (2012). Arbitrage in skew Brownian motion models. Insur. Math. Econom..

[26] Hussain J., Soomro M.A., Dahri S.A., Memon K.N., Bano M., Awwad F.A., Ismail E.A.A., Ahmad H. (2024). A study of maximizing skew Brownian motion with applications to option pricing. J. Radiat. Res. Appl. Sci..

[27] Dassios M., Zhang J. (2022). First Hitting Time of Brownian Motion on Simple Graph with Skew Semiaxes. Methodol. Comput. Appl. Probab..

[28] Atar R., Cohen A. (2019). Serve the shortest queue and Walsh Brownian motion. Ann. Appl. Probab..

[29] Salminen P., Stenlund D. (2021). On occupation times of one-dimensional diffusions. J. Theoret. Probab..

[30] Itô K., McKean H.P. (1974). Diffusion Processes and Their Sample Paths.

[31] Borodin A.N., Salminen P. (2015). Handbook of Brownian Motion—Facts and Formulae.

[32] Kac M. (1951). On some connections between probability theory and differential and integral equations. Proceedings of the Second Berkeley Symposium on Mathematical Statistics and Probability.

[33] Stenlund D. (2022). On the connection between Stirling numbers and Bessel numbers. Electron. J. Combin..

[34] Yang S.L., Qiao Z.K. (2011). The Bessel numbers and Bessel matrices. J. Math. Res. Expo..

[35] Graham R.L., Knuth D.E., Patashnik O. (1994). Concrete Mathematics.

